# Supplementary material for: A Critical View of the Application of the APEX Software (Aqueous Photochemistry of Environmentally-Occurring Xenobiotics) to Predict Photoreaction Kinetics in Surface Freshwaters
Source: Molecules. 2019 Dec 18;25(1):9. doi: 10.3390/molecules25010009 (PMC7017383; doi:10.3390/molecules25010009)
Supplement: Supplementary file 1 [file molecules-25-00009-s001.zip › 3.2.4_gcc-4.4.0.zip!/3.2.4_gcc-4.4.0/3.2.4_gcc-4.4.0/share/octave/packages/bim-1.0.0/doc/tutorial.html]

This is a short example on how to use bim to solve a DAR problem.  
The data for this example can be found in the doc directory inside the
bim installation directory.

 **Create the mesh and precompute the mesh properties**   
The geometry of the domain was created using gmsh and is stored in the file fiume.geo

```
[mesh] = msh2m_gmsh("fiume","scale",1,"clscale",.1);
[mesh] = bim2c_mesh_properties(mesh);
```

 **Construct an initial guess**  
For a linear problem only the values at boundary nodes are actually relevant  

```
xu     = mesh.p(1,:);
yu     = mesh.p(2,:);
nelems = columns(mesh.t);
nnodes = columns(mesh.p);
uin    = 3*xu;
```

 **Set the coefficients for the problem:
-div ( \alpha \gamma ( \eta \nabla u - \beta u ) )+ \delta \zeta u = f g**  

```
epsilon = .1;
alfa    = ones(nelems,1);
gamma   = ones(nnodes,1);
eta     = epsilon*ones(nnodes,1);
beta    = xu+yu;
delta   = ones(nelems,1);
zeta    = ones(nnodes,1);
f       = ones(nelems,1);
g       = ones(nnodes,1);
```

 **Construct the discretized operators**  

```
AdvDiff = bim2a_advection_diffusion(mesh,alfa,gamma,eta,beta);
Mass    = bim2a_reaction(mesh,delta,zeta);
b       = bim2a_rhs(mesh,f,g);
A       = AdvDiff + Mass;
```

 **To Apply Boundary Conditions, partition LHS and RHS**  
The tags of the sides are assigned by gmsh

```
Dlist = bim2c_unknowns_on_side(mesh, [8 18]); 	   ## DIRICHLET NODES LIST
Nlist = bim2c_unknowns_on_side(mesh, [23 24]); 	   ## NEUMANN NODES LIST
Nlist = setdiff(Nlist,Dlist);
Fn    = zeros(length(Nlist),1);           	   ## PRESCRIBED NEUMANN FLUXES
Ilist = setdiff(1:length(uin),union(Dlist,Nlist)); ## INTERNAL NODES LIST
```

```
Add = A(Dlist,Dlist);
Adn = A(Dlist,Nlist); ## shoud be all zeros hopefully!!
Adi = A(Dlist,Ilist); 

And = A(Nlist,Dlist); ## shoud be all zeros hopefully!!
Ann = A(Nlist,Nlist);
Ani = A(Nlist,Ilist); 

Aid = A(Ilist,Dlist);
Ain = A(Ilist,Nlist); 
Aii = A(Ilist,Ilist); 

bd = b(Dlist);
bn = b(Nlist); 
bi = b(Ilist); 

ud = uin(Dlist);
un = uin(Nlist); 
ui = uin(Ilist);
```

 **Solve for the displacements**  

```
temp = [Ann Ani ; Ain Aii ] \ [ Fn+bn-And*ud ; bi-Aid*ud];
un   = temp(1:length(un));
ui   = temp(length(un)+1:end);
```

 **Compute the fluxes through Dirichlet sides**  

```
Fd = Add * ud + Adi * ui + Adn*un - bd;
```

 **Compute the gradient of the solution**   

```
[gx, gy] = bim2c_pde_gradient(mesh,u);
```

 **Compute the internal Advection-Diffusion flux**  

```
[jxglob,jyglob] = bim2c_global_flux(mesh,u,alfa,gamma,eta,beta);
```

 **Save data for later visualization**  

```
fpl_dx_write_field("dxdata",mesh,[gx gy],"Gradient",1,2,1);
```
